# Supplementary figures and images for: Mining Key Drought-Resistant Genes of Upland Cotton Based on RNA-Seq and WGCNA Analysis
Source: Plants (Basel). 2025 May 8;14(10):1407. doi: 10.3390/plants14101407 (PMC12114639; doi:10.3390/plants14101407)

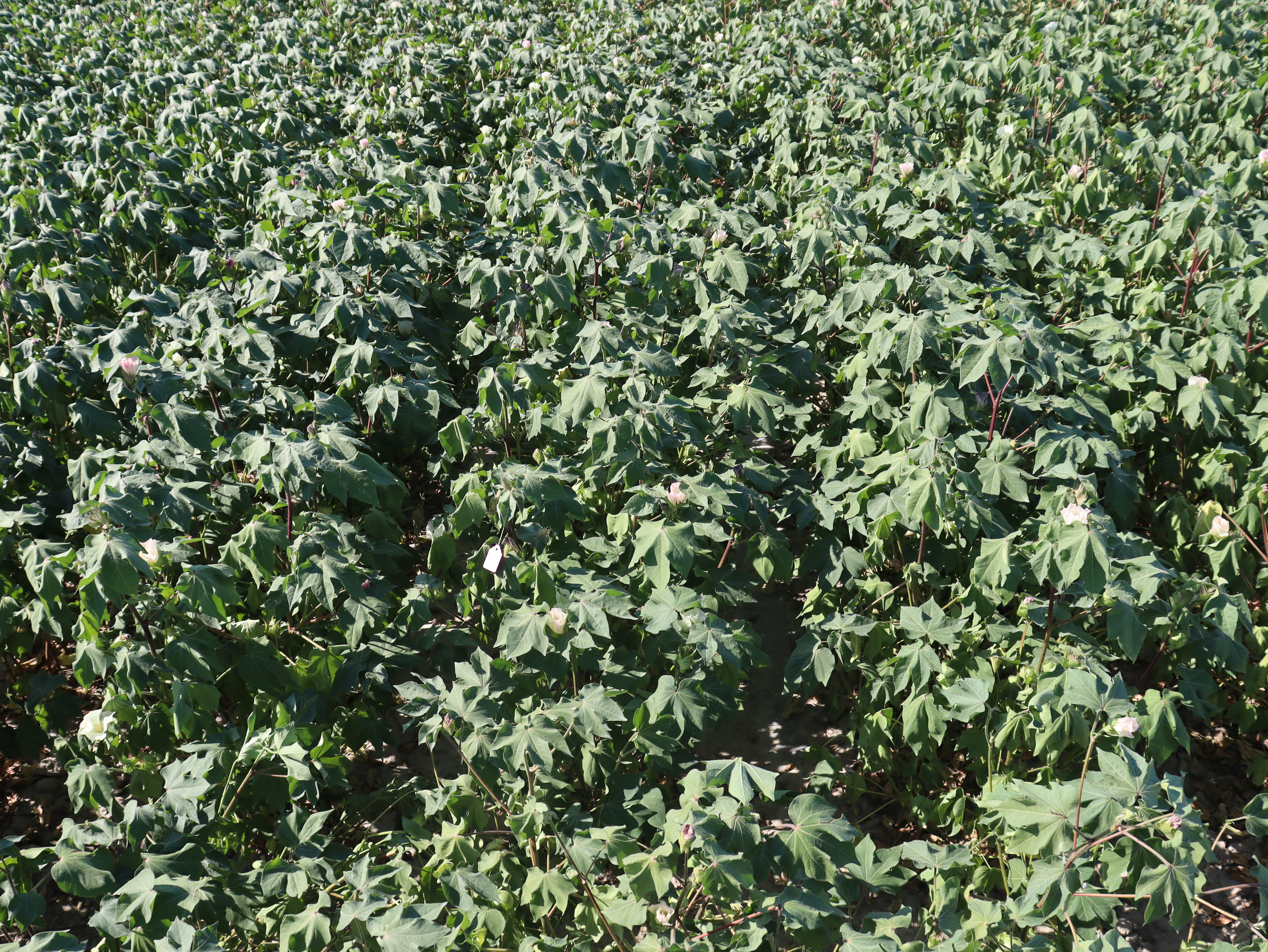

Supplement: Supplementary file 1 [file plants-14-01407-s001.zip › Supplementary Materials/Figure S1.JPG]

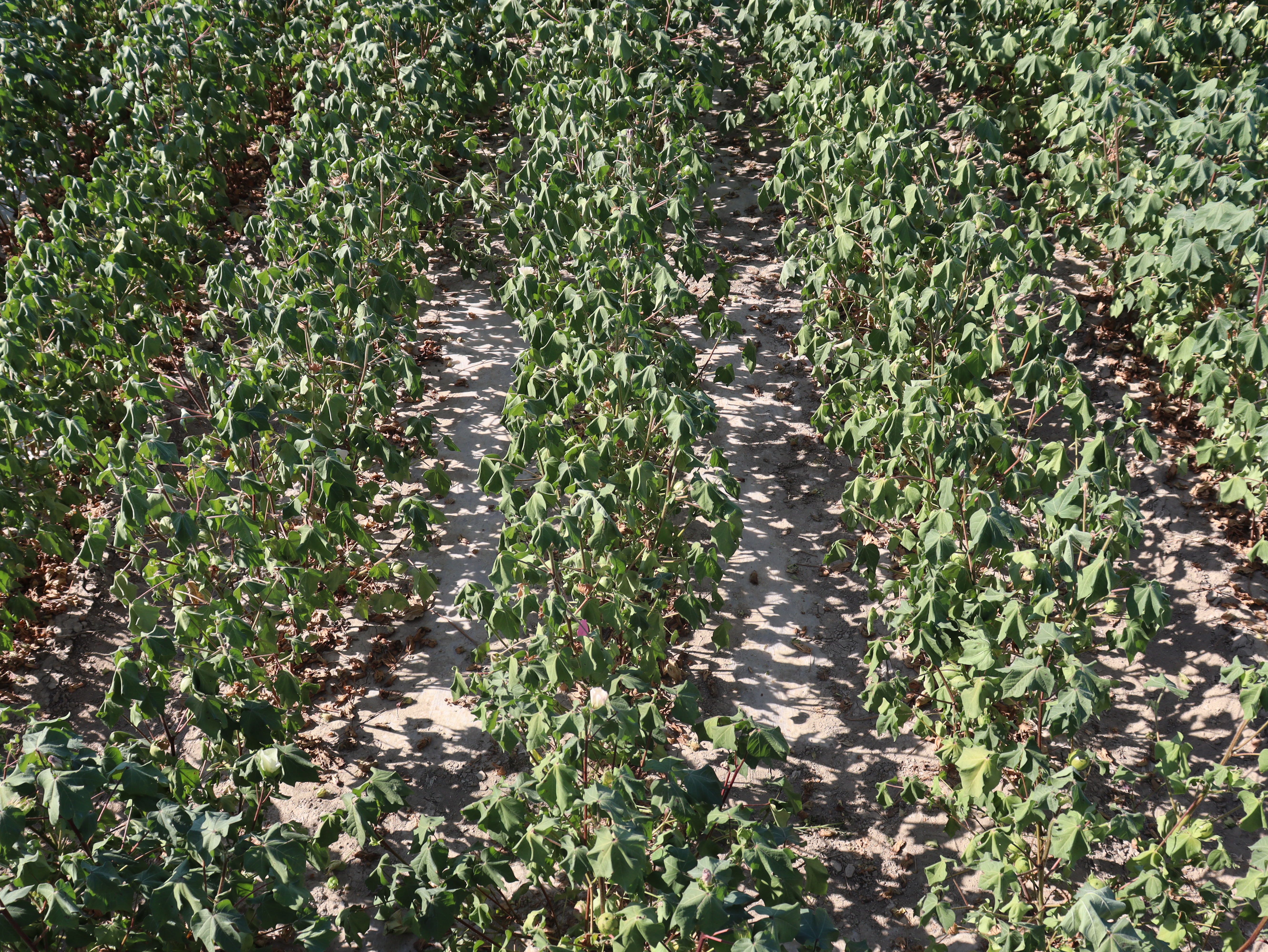

Supplement: Supplementary file 1 [file plants-14-01407-s001.zip › Supplementary Materials/Figure S2.JPG]
